# Enrichment of apolipoprotein A-IV and apolipoprotein D in the HDL proteome is associated with HDL functions in diabetic kidney disease without dialysis

**DOI:** 10.1186/s12944-020-01381-w

**Published:** 2020-09-14

**Authors:** Monique F. M. Santana, Aécio L. A. Lira, Raphael S. Pinto, Carlos A. Minanni, Amanda R. M. Silva, Maria I. B. A. C. Sawada, Edna R. Nakandakare, Maria L. C. Correa-Giannella, Marcia S. Queiroz, Graziella E. Ronsein, Marisa Passarelli

**Affiliations:** 1 Laboratório de Lípides (LIM-10), Hospital das Clínicas (HCFMUSP) da Faculdade de Medicina da Universidade de São Paulo, Av. Dr. Arnaldo 455, room 3305; CEP, São Paulo, 01246-000 Brazil; 2 Centro Universitário CESMAC, Maceio, Alagoas Brazil; 3 Faculdade Israelita de Ciências da Saúde Albert Einstein, Hospital Israelita Albert Einstein (HIAE), São Paulo, Brazil; 4 Departamento de Bioquímica, Instituto de Química, Universidade de São Paulo, São Paulo, Brazil; 5 Programa de Pós-Graduação em Medicina, Universidade Nove de Julho, São Paulo, Brazil; 6 Laboratório de Carboidratos e Radioimunoensaio (LIM 18), Hospital das Clínicas (HCFMUSP) da Faculdade de Medicina da Universidade de São Paulo, São Paulo, Brazil

**Keywords:** Diabetic kidney disease, Advanced glycation, Carbamoylation, HDL, Apolipoprotein A-IV, Apolipoprotein D, Proteomics, Atherosclerosis

## Abstract

**Background and aims:**

Diabetic kidney disease (DKD) is associated with lipid derangements that worsen kidney function and enhance cardiovascular (CVD) risk. The management of dyslipidemia, hypertension and other traditional risk factors does not completely prevent CVD complications, bringing up the participation of nontraditional risk factors such as advanced glycation end products (AGEs), carbamoylation and changes in the HDL proteome and functionality. The HDL composition, proteome, chemical modification and functionality were analyzed in nondialysis subjects with DKD categorized according to the estimated glomerular filtration rate (eGFR) and urinary albumin excretion rate (AER).

**Methods:**

Individuals with DKD were divided into eGFR> 60 mL/min/1.73 m^2^ plus AER stages A1 and A2 (*n* = 10) and eGFR< 60 plus A3 (*n* = 25) and matched by age with control subjects (eGFR> 60; *n* = 8).

**Results:**

Targeted proteomic analyses quantified 28 proteins associated with HDL in all groups, although only 2 were more highly expressed in the eGFR< 60 + A3 group than in the controls: apolipoprotein D (apoD) and apoA-IV. HDL from the eGFR< 60 + A3 group presented higher levels of total AGEs (20%), pentosidine (6.3%) and carbamoylation (4.2 x) and a reduced ability to remove ^14^C-cholesterol from macrophages (33%) in comparison to HDL from controls. The antioxidant role of HDL (lag time for LDL oxidation) was similar among groups, but HDL from the eGFR< 60 + A3 group presented a greater ability to inhibit the secretion of IL-6 and TNF-alpha (95%) in LPS-elicited macrophages in comparison to the control group.

**Conclusion:**

The increase in apoD and apoA-IV could contribute to counteracting the HDL chemical modification by AGEs and carbamoylation, which contributes to HDL loss of function in well-established DKD.

## Background

In diabetes mellitus (DM), abnormal kidney function is one of the most frequent complications and is the leading cause of end-stage kidney disease. In addition, kidney function impairment increases the risk of cardiovascular disease (CVD), which is the major cause of mortality in both type 1 (DM1) and type 2 DM (DM2) [1]. Apart from traditional risk factors that are commonly observed in DM, including dyslipidemia, hypertension and other components of the metabolic syndrome, untraditional risk factors – advanced glycation, carbamoylation and oxidation – contribute to macrovascular disease in diabetic kidney disease (DKD) [2–4].

In DKD, the reduction in kidney function represented by the diminished estimated glomerular filtration rate (eGFR) is not invariable accompanied by the same extension by elevation in the urinary albumin excretion rate (AER) [5]. In fact, many individuals with DKD with a marked reduction in eGFR can still present normal (A1 stage) or slightly reduced AER (A2 stage), and in some cases, people in A2 stage can revert to A1 [6]. The incidence of CVD is positively related to the reduction in eGFR as well as AER, and both parameters have additive effects on CVD risk in any stage of abnormal kidney function [7].

Advanced glycation end products (AGEs) are prevalent in DM and in chronic kidney disease (CKD) due to hyperglycemia, oxidative stress, inflammation and detoxification failure of intermediate compounds of the glycation reaction. AGEs are independent predictors of CVD risk by impairing reverse cholesterol transport (RCT) and lipid metabolism, inducing inflammation and altering vasodilation [8–10]. In addition, the reaction of isocyanic acid – derived from urea or from myeloperoxidase activity – with proteins leads to protein carbamoylation, which is also related to atherogenesis [4]. Diet and tobacco contribute as unpredictable sources of thiocyanate that favor carbamoylation and AGEs, increasing the body pool of carbamoylated and glycated macromolecules.

The reduction in high-density lipoprotein (HDL) cholesterol in plasma is a hallmark of DM, although HDL dysfunction is also considered to have an important role in CVD morbidity and mortality. This is especially reputable when analyzing clinical trials where the increment in HDL cholesterol did not contribute to CVD risk improvement. HDL are antiatherogenic particle that mediate the removal of excess cholesterol from arterial wall macrophages, allowing its trafficking to the liver and excretion in feces by the RCT. In addition, HDL exerts several other atheroprotective actions, including antioxidant, anti-inflammatory, vasodilation and antiaggregant activities, and improves glucose tolerance and insulin sensitivity. HDL is a cargo lipoprotein that transports many proteins, microRNAs and other molecules that are able to control metabolism in different tissues and in the arterial wall [11].

The HDL proteome that follows its complexity and functionality has been analyzed in CKD associated or not with DM, but in most of the studies, the individuals were on dialysis, which may have influenced the results. In addition, the stratification of subjects with CKD by eGFR or AER alone may not reflect changes in the HDL proteome and function that take place in an unusual evolution of chronic disease, typically in DKD. The hypothesis of this study is that the HDL proteome and its chemical modification may influence the functionality of this lipoprotein and its role in CVD in DKD without dialysis. Then, the HDL proteome, composition and modification by advanced glycation and carbamoylation, together with its functionality were analyzed in nondialysis subjects with DKD categorized according to the eGFR and AER in comparison to age-matched control subjects. The HDL proteome was enriched in apoD and apoA-IV, and HDL was modified by advanced glycation and carbamoylation, according to the reduction in eGFR and increased AER. The ability of HDL to remove cellular cholesterol was reduced in DKD with eGFR < 60 mL/min/1.73 m^2^ plus A3, although its antioxidant activity was preserved and its capacity to prevent inflammation in macrophages was even increased. This may be related to the increment of HDL in apoA-IV that exerts antiatherogenic actions and apoD counteracting HDL loss of function that is reported in CKD.

## Material and methods

Subjects with DM2 with DKD were recruited at the Hospital das Clínicas da Faculdade de Medicina da Universidade de São Paulo. The inclusion criteria was based on the diagnosis of DM2 for at least 10 years and on the classification of chronic kidney disease according to the loss in the glomerular filtration rate (eGFR; < 60 mL/min/1.73 m^2^) and enhanced urinary albumin excretion rate [AER; A1: < 30 mg/g creatinine; A2: 30–300 mg/g creatinine and A3: > 300 mg/g creatinine] for at least 3 months [12]. HbA1c values were between 7 and 10%, avoiding greater variations in DM control and carbonyl stress due to hyperglycemia.

Healthy control individuals matched by age were selected at Faculdade de Medicina da Universidade de São Paulo. All participants were properly informed about the procedures and the study and signed an informed written consent form that was previously approved by The Ethical Committee for Human Research Protocols of the Clinical Hospital (#15024), in accordance with the Declaration of Helsinki. Subjects on dialysis, with other chronic diseases, rapid loss in eGFR (> 3 mL/min/year), refractory hypertension, BMI < 18.5 kg/m^2^, current smokers or those who abused alcohol were not included. Eighty percent of subjects with DKD were using insulin, and 70% were on statins and beta-blockers. Angiotensin-converting-enzyme inhibitors (ACEi) and metformin were used for A1 and A2 subjects only, and angiotensin II receptor blockers (ARBs) were used by groups A1 (39%), A2 (50%) and A3 (36%). Erythropoietin was utilized by the A2 (7%) and A3 (12%) groups.

Blood was drawn after overnight fasting, and HbA1c was determined by high-performance liquid chromatography (HPLC). Plasma was immediately isolated from the same sample in a refrigerated centrifuge (4 °C). Glycemia, triglycerides (TG), total cholesterol (TC), HDL cholesterol (HDLc), fructosamine, creatinine and urea were determined in plasma by enzymatic techniques after overnight fasting and albumin in 24 h urine. Individuals with DKD were categorized according to eGFR above 60 mL/min/1.73 m^2^ plus AER stages A1 (< 30 mg/g creatinine) and A2 (30–300 mg/g creatinine) and eGFR below 60 mL/min/1.73 m^2^ plus stage A3 (> 300 mg/g creatinine). Control subjects presented eGFR above 60 mL/min/1.73 m^2^ plus A1.

### Isolation of lipoproteins

Venous blood samples were drawn after overnight fasting, and plasma was immediately isolated in a refrigerated centrifuge. Preservatives were added to the plasma, and the density was adjusted with bromide potassium to 1.21 g/mL. Low-density (LDL; d = 1.019–1.063 g/mL) and high-density lipoprotein (HDL; d = 1.063–1.21 g/mL) were isolated from plasma by discontinuous density gradient ultracentrifugation (100,000 *g*, 24 h, 4 °C, Sw40 rotor; Beckman ultracentrifuge). Samples were dialyzed against phosphate-buffered saline containing EDTA (PBS).

### HDL composition in lipids

The amount of lipids in HDL was determined by enzymatic techniques [TC and TG; Labtest diagnóstica S.A., Minas Gerais, Brazil; phospholipids; Randox Laboratorier LTD. Crumlin, Co., Antrem, United Kingdom].

### Determination of total AGEs and pentosidine in HDL

The contents of total AGEs and pentosidine were determined in isolated HDL by fluorescence measurement (Synergy HT Multi-Mode Microplate Reader, SpectraMax M5). Samples were excited at a wavelength of 370 nm, and the fluorescence emitted was 440 nm and 378 nm for total AGEs and pentosidine, respectively [13].

### Determination of HDL carbamoylation

HDL carbamoylation was determined in the isolated lipoprotein by ELISA (− STA-877 Protein Carbamylation Sandwich ELISA; Cell Biolabs Inc., San Diego, CA, USA).

### Determination of carboxymethyllysine in plasma

Carboxymethyllysine (CML) was determined in plasma by ELISA (Circulex CML, Woburn, MA, USA).

### Proteolytic digestion of HDL

The HDL protein concentration was determined by the Bradford assay (Bio-Rad, Hercules, CA, USA). Ten micrograms of HDL protein was solubilized in 100 mM ammonium bicarbonate, dithiothreitol, and iodoacetamide, following digestion with trypsin (1:40, w/w Promega, Madison, WI, USA) for 4 h at 37 °C. Trypsin was further added to the samples (1:50, w/w HDL), and incubation was performed overnight at 37 °C. Samples were desalted using solid phase extraction (Oasis PRIME HLB SPE column; Waters) after acidic hydrolysis with 2% trifluoroacetic acid, dried and kept frozen at − 80 °C until MS analyses. Prior to MS analysis, samples were resuspended in 0.1% formic acid (final protein concentration of 25 ng/μL).

Angiotensin peptide (DRVYIHPFHL, 0.2 pmol/μL) spiked in each sample was used as a global internal standard to control the robustness of the PRM methodology. Variability in the integrated peptide area was monitored across 87 injections, and low variance was obtained with a CV of 13%.

### Targeted proteomic analyses

Digested HDL proteins (50 ng protein) were quantified by parallel reaction monitoring (PRM), as previously described [14]. Briefly, an Easy-nLC 1200 UHPLC (Thermo Scientific, Bremen, Germany) was used for peptide separation. Each sample was loaded onto a trap column (nanoViper C18, 3 μm, 75 μm × 2 cm, Thermo Scientific), and after, the trapped peptides were eluted onto a C18 column (nanoViper C18, 2 μm, 75 μm × 15 cm, Thermo Scientific). Acquisition of the data was performed in an Orbitrap Fusion Lumos mass spectrometer (Thermo Scientific, Bremen, Germany) using a nanospray Flex NG ion source (Thermo Scientific, Bremen, Germany). A scheduled (3-min window) inclusion list containing m/z of precursor peptides of interest and corresponding retention times was generated using Skyline software [15]. MS proteomics data have been deposited to the Mass Spectrometry Interactive Virtual Environment (MassIVE) with access via ftp://MSV000085663@massive.ucsd.edu and doi:10.25345/C5VJ15 (username: mfmsproteomic2020 password: 03Ja/94na).

### Selection of HDL peptides for targeted quantification

PRM methodology was assembled using data derived from shotgun proteomics analyses as previously described [14]. Ninety-one proteins were identified, but this number was reduced to 47 proteins after eliminating proteins that could be potential contaminants or were in low abundance (keratin, proteins with < 2 unique peptides and peptides with high interfering signal). Peptides susceptible to ex vivo modification (e.g., methionine-containing peptides) were also avoided, and only peptides satisfactorily detected (with a good chromatographic peak, containing at least 4 coeluted transitions, and with mass error < 10 ppm) were included in the final analysis. After exclusion criteria, 28 proteins remained. For each protein quantification, a surrogate peptide was chosen by first selecting a peptide pair with the best Pearson’s correlation coefficient, followed by empirically selecting the final peptide based on a good chromatographic peak. Quantification was performed using the sum of peak areas obtained for each transition of each surrogate peptide, and at least 4 transitions per peptide were used. The 28 surrogate peptides chosen for HDL proteins are highlighted in Table 1.

**Table 1:** Proteins found in the HDL proteome of controls and DKD subjects. 28 proteins were quantified by PRM Targeted Proteomic in HDL from controls and DKD subjects

Gene name	Protein name	Peptide sequence
*SERPINA1*	Alpha-1 antitrypsin (A1AT)	LSITGTYDLK
*AMBP*	AMBP protein (AMBP)	AFIQLWAFDAVK
*APOA1*	Apolipoprotein A-I (apoA-I)	DYVSQFEGSALGK
*APOA2*	Apolipoprotein A-II (apoA-II)	SPELQAEAK
*APOA4*	Apolipoprotein A-IV (apoA-IV)	LTPYADEFK
*APOB*	Apolipoprotein B-100 (apoB100)	SVSLPSLDPASAK
*APOC1*	Apolipoprotein C-I (apoC-I)	EFGNTLEDK
*APOC2*	Apolipoprotein C-II (apoC-II)	ESLSSYWESAK
*APOC3*	Apolipoprotein C-III (apoC-III)	GWVTDGFSSLK
*APOC4*	Apolipoprotein C-IV (apoC-IV)	AWFLESK
*APOD*	Apolipoprotein D (apoD)	NILTSNNIDVK
*APOE*	Apolipoprotein E (apoE)	VQAAVGTSAAPVPSDNH
*APOF*	Apolipoprotein F (apoF)	SGVQQLIQYYQDQK
*APOH*	Apolipoprotein H (apoH)	EHSSLAFWK
*APOL1*	Apolipoprotein L (apoL)	VAQELEEK
*APOM*	Apolipoprotein M (apoM)	DGLCVPR
*C3*	C3 complement (CO3)	DFDFVPPVVR
*CETP*	Cholesterol ester transfer protein (CETP)	ASYPDITGEK
*CLU*	Clusterin (Clus)	LFDSDPITVTVPVEVSR
*LCAT*	Lecithin cholesterol acyltransferase (LCAT)	SSGLVSNAPGVQIR
*PON1*	Paraoxonase arylesterase 1 (PON1)	IQNILTEEPK
*PON3*	Paraoxonase lactonase 3 (PON3)	STVEIFK
*PLTP*	Phospholip transfer protein (PLTP)	AGALQLLLVGDK
*PCYOX1*	Prenilcystein oxidase 1 (PCYOX)	LFLSYDYAVK
*SAA1*	Serum amyloid A-I (SAA1)	GPGGVWAAEAISDAR
*SAA4*	Serum amyloid 4 (SAA4)	FRPDGLPK
*TTR*	Transtirretin (TTHY)	GSPAINVAVHVFR

### Acetylation of LDL

LDL was acetylated as previously described by Basu et al. [16]. Samples were extensively dialyzed before incubation with macrophages.

### Measurement of ^14^C-cholesterol efflux

This study was approved by the Institutional Animal Care and Research Advisory Committee (#1015/2018) and was performed following the U.S. National Institutes of Health Guide for the Care and Use of Laboratory Animals. C57BL/6 J mice were housed in a conventional animal facility at 22 ± 2 °C under a 12-h light/dark cycle with free access to commercial chow (Nuvilab CR1, São Paulo, Brazil) and drinking water. Bone marrow-derived cells were isolated from male, 6-week-old mice, and macrophages were differentiated [17]. Briefly, tissues from the femur and tibias were cleaned and isolated at the knee joint. A needle size 26 and ½ and a 20-mL syringe filled with bone marrow medium (low-glucose DMEM with 0.8% penicillin/streptomycin, 10% heat-inactivated fetal calf serum, and 10% L929 cell-conditioned medium) were utilized to cut the end of each bone and to expel the bone marrow from both ends of the bones. Bone marrow was aspirated and expelled by utilizing a needle size 18 and ½ attached to a 20-mL syringe. Cells were centrifuged (6 min, 1000 rpm at room temperature), resuspended in bone marrow medium, plated in culture dishes, and incubated for 5 days at 37 °C under 5% (v/v) CO2. Then, the medium was changed to low-glucose DMEM containing 1% penicillin/streptomycin + 10% heat-inactivated fetal calf serum.

Bone marrow-derived macrophages (BMDMs) were overloaded with acetylated LDL (50 μg/mL DMEM) and ^14^C-cholesterol (0.3 μCi/mL) for 48 h. HDL particles from controls and subjects with DKD (50 μg/mL) were utilized as cholesterol acceptors in 6-h incubations, and the percentage of cholesterol efflux was calculated as ^14^C-cholesterol in the media/^14^C-cholesterol in the medium +^14^C-cholesterol in cells × 100. Control incubations were performed in the presence of DMEM containing fatty-acid-free albumin (FAFA) in the absence of HDL, and the results were subtracted from those obtained in the presence of HDL, as previously described [8].

### Measurement of HDL antioxidant activity

The ability of HDL from controls and individuals with DKD to inhibit LDL oxidation was determined by incubation of LDL (40 μg/mL) isolated from a unique healthy plasma donor with CuSO_4_ solution (1 mL; final concentration 10 μmol/L) in the presence of HDL (80 μg/mL). Lipoproteins were dialyzed against PBS without EDTA prior to incubation. The absorbance at 234 nm was continuously monitored every 3 min for 4 h, and the lag time phase for LDL oxidation (min) and the maximum ratio of conjugated diene formation were calculated [18].

### Measurement of HDL anti-inflammatory activity

BMDMs were isolated and cultured as described above and then overloaded with acetylated LDL (50 μg/mL DMEM) and treated for 24 h with HDL (50 μg/mL DMEM) from controls and subjects with DKD. After washing, macrophages were incubated with lipopolysaccharide (LPS; 1 μg/mL DMEM) for 24 H*. medium* was collected, and the amount of TNF-alpha and interleukin-6 (IL-6) was determined by ELISA (R&D System-Duo Set, Minneapolis, EUA) [9].

### Statistical analysis

Statistical analysis was performed using the GraphPad Prism 5 program (GraphPad Software, Inc. 2007). Comparisons were made by the Kruskal-Wallis test with the Holm-Sidak posttest, Mann-Whitney or Student’s t test and Spearman’s linear correlation as appropriate. A value of *P* < 0.05 was considered statistically significant.

## Results

Anthropometric and biochemical data of controls and subjects with DKD are depicted in Table 2. In the eGFR < 60 + A3 group, there was a greater predominance of male individuals (72%) compared to the control (50%) and eGFR> 60 + A1 and A2 (30%) groups. Age was similar among groups as well as the time of DM when comparing both DKD groups. BMI and CVD history were higher in the eGFR > 60 + A1 and A2 group. The measurement of traditional variables of glycemic control showed that fructosamine and HbA1c levels were higher in the DM groups than in controls. On the other hand, the major circulating AGE species in plasma, CML, did not reach statistical significance among the groups. Total cholesterol (TC) was lower in the eGFR > 60 + A1 and A2 group than in the control group.

**Table 2:** Anthropometric and clinical data of control subjects and individuals with DKD categorized according to the estimated glomerular filtration rate (eGFR; mL/min/1.73 m^2^) and albumin excretion rate (AER; A1 = normoalbuminuria, A2 = microalbuminuria; A3 = macroalbuminuria)

	Control eGFR > 60	DKD eGFR > 60 A1 + A2	DKD eGFR < 60 A3
n (F/M)	8 (4/4)	10 (7/3)	25 (7/18)
Age (years)	68.5 (58–84)	68 (53–75)	69 (55–87)
eGFR (mL/min/1.73m^2^)	84.5 (63–102)	82.0 (61–131)	25 (10–46)^#^
AER (mg/g creatinine)	–	9.5 (2.4–114)	1128 (317–6430)^@^
Time of DM (years)	–	14 (4–26)	18 (6–30)
CVD history (%)	–	2 (20%)	11 (44%)
BMI (kg/m^2^)	24.9 (21–29)	33 (25–45) *	27 (21–38)
HbA1c (%)	5.6 (5.0–6.0)	8.4 (7.0–8.0)^#^	8.3 (6.0–10.0) ^#^
Fructosamine (μmol/L)	219 (205–262)	308 (158–428)^&^	331 (329–506) ^&^
TC (mg/dL)	208 (155–290)	144 (114–186)^$^	154 (92–313)
TG (mg/dL)	111 (71–362)	88 (64–151)	148 (78–329)
HDLc (mg/dL)	49 (33–87)	46 (35–92)	40 (21–140)
CML (μg/mL)	0.5 (0.3–1.0)	1.7 (0.5–3.4)	1.0 (0.7–7.3)

*BMI* body mass index, *HbA1c* glycated hemoglobin, *TC* total cholesterol, *TG* triglycerides, *HDLc* HDL cholesterol, *CML* carboxymethyl-lysine**.** Results were compared by the Kruskal-Wallis test with Holm-Sidak posttest (median - range); * *p* < 0.004; ^#^ *p* < 0.0001; ^&^ *p* < 0.001 and ^$^ *p* < 0.006 in comparison to Control GFR > 60, and by Mann- Whitney test; @ *p* < 0.001 in comparison to eGFR > 60 A1 + A2

**Fig. 1:**
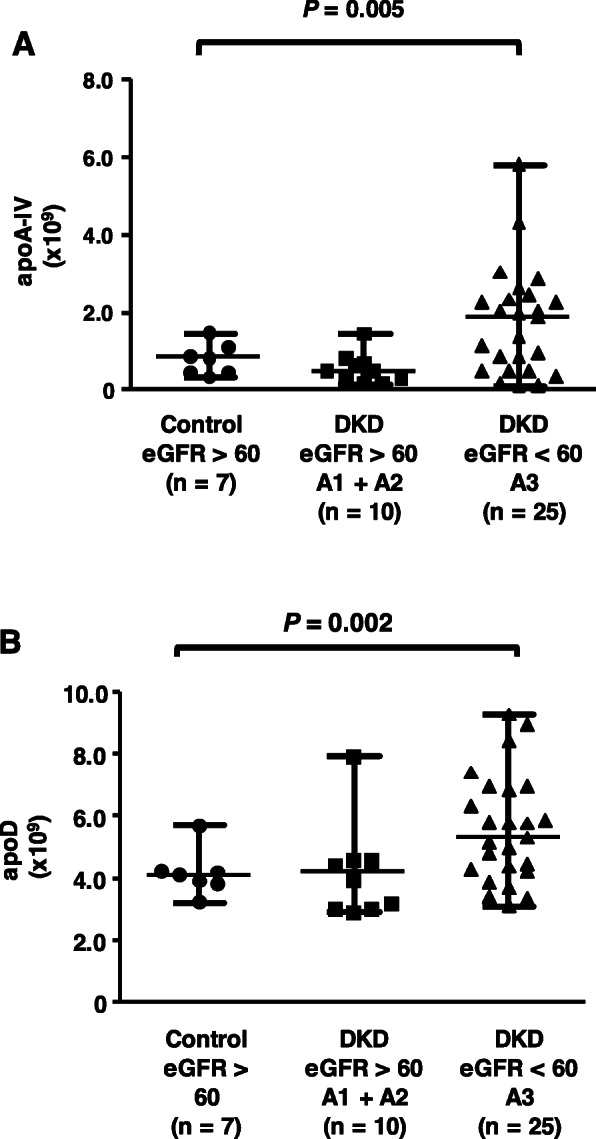
Significant proteins in the CKD-HDL proteome. Differentially expressed proteins in the CKD-HDL proteome included apoA-IV and apoD (panels **a**-**b**). Comparisons were made by the Kruskal-Wallis test with the Holm-Sidak posttest

Proteomic analysis was performed to analyze the protein cargo of HDL and its possible implication in HDL functionality. Twenty-eight proteins associated with HDL were quantified by PRM using nanoscale liquid chromatography coupled with mass spectrometry (Nano-LC/MS/MS) **(**Table 1**)**. Of these, 2 were more highly expressed in the eGFR < 60 + A3 group than in the control group: apoA-IV **(**Fig. 1 panel a) and apoD (Fig. 1 panel b).

**Fig. 2:**
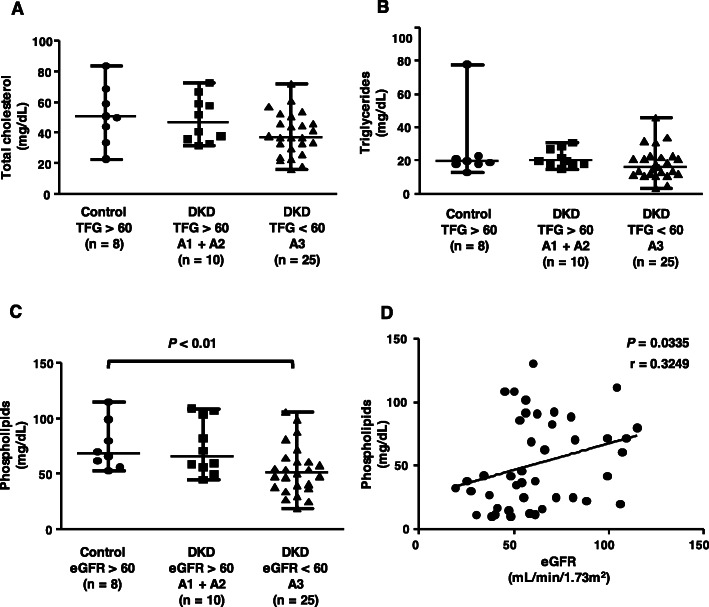
HDL composition. The HDL content of total cholesterol, triglycerides and phospholipids was determined by colorimetric enzymatic methods (panels **a**-**c**). The results were compared by the Kruskal-Wallis test with the Holm-Sidak posttest. Association between HDL-phosphoplipids with eGFR (panel **d**) was performed by Spearman’s correlation

The HDL composition (Fig. 2) was addressed to verify possible changes in lipid content that may modulate the HDL ability to remove cell cholesterol and inhibit oxidation and inflammation. The HDL content in TC (Fig. 2 panel a) and TG (Fig. 2 panel b) was similar among all groups. There was a decrease in phospholipids (Fig. 2 panel c) in the HDL of the eGFR group < 60 + A3 compared to the control group and a positive correlation was observed between HDL-phospholipids (Fig. 2 panel d) with the eGFR.

**Fig. 3:**
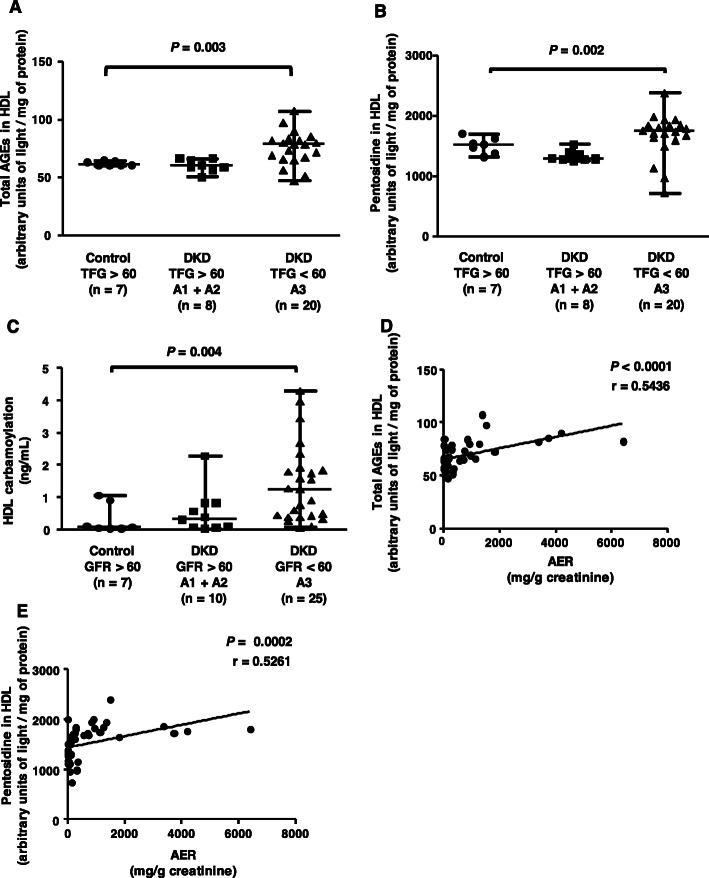
HDL modification by advanced glycation and carbamoylation. The amount of total AGEs (panel **a**) and pentosidine (panel **b**) was determined in HDL by measuring the absorbance in the fluorescence range at 440 nm (total AGEs) and 378 nm (pentosidine) and carbamoylation (panel **c**) by ELISA. The results were compared by the Kruskal-Wallis test with the Holm-Sidak posttest. Associations between HDL chemical modification and AER (panels **d** and **e**) were performed by Spearman’s correlation

Chemical modification by glycation and carbamoylation was determined in isolated HDL. Total AGEs (Fig. 3 panel a) and pentosidine (Fig. 3 panel b) were higher in HDL isolated from individuals with eGFR < 60 + A3 (20 and 6.3%, respectively) than in the control group. Although the values of HbA1c and fructosamine were similar between groups, carbonyl stress as a function of albuminuria and eGFR < 60 can be attributed to the renal changes that accompany macroalbuminuria and are reflected in the lower detoxification of glycation reaction precursors and greater oxidative stress. The modification of HDL by isocyanic acid, which reflects uremic stress and induces HDL carbamoylation, was greater in the group with eGFR < 60 + A1 than in the control group (Fig. 3 panel c). A positive correlation was observed between total AGEs (Fig. 3, panel d) and pentosidine in HDL **(**Fig. 3 panel e) with AER.

**Fig. 4:**
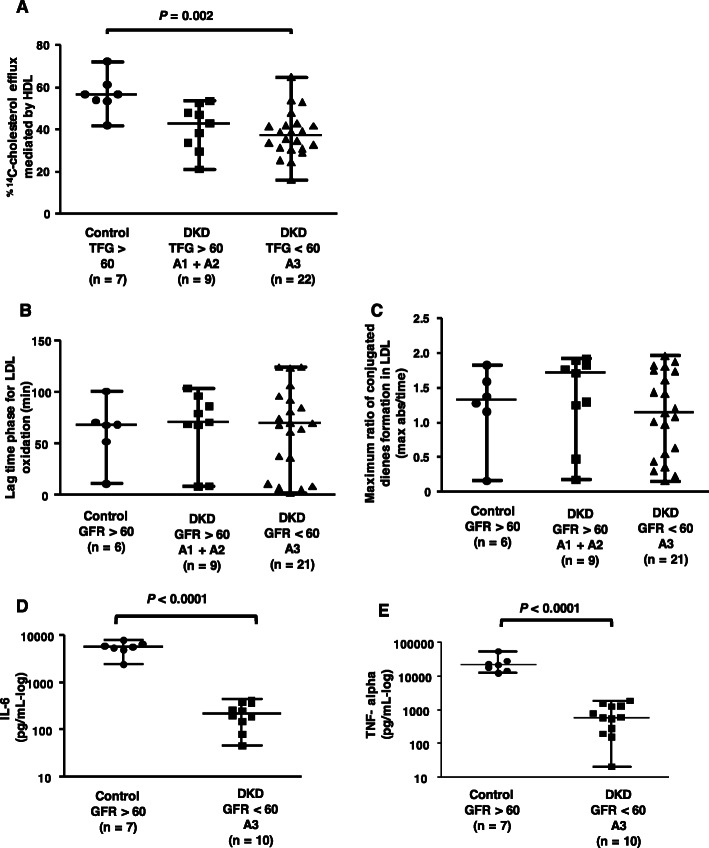
HDL functionality. (Panel **a**) Cholesterol efflux: HDL was isolated from subjects with DKD and controls and utilized as an acceptor of cellular cholesterol. Bone marrow-derived macrophages (BMDMs) overloaded with acetylated LDL and ^14^C-cholesterol were incubated with 50 μg of HDL/mL of medium for 6 h. Cholesterol efflux was determined after measuring the radioactivity in the culture medium and that remaining in cells, which was calculated as ^14^C-cholesterol in the medium/^14^C-cholesterol in the medium +^14^C-cholesterol in cells × 100. Control incubations were performed in the presence of DMEM/FAFA in the absence of HDL, and the results were subtracted from those obtained in the presence of HDL. (Panels **b** and **c**) Antioxidant activity: The lag time (panel **b**) and the maximum rate of LDL oxidation (panel **c**) were determined in incubations with LDL (40 μg of protein) isolated from a healthy donor with CuSO_4_ solution and HDL from DKD or controls (80 μg of protein). (Panels **d** and **e**) Anti-inflammatory activity: BMDMs overloaded with acetylated LDL (50 μg/mL) were treated with HDL (50 μg/mL) for 24 h. After washing, cells were treated with LPS (1 μg/mL) for 24 h, and interleukin-6 (IL-6, panel **d**) and TNF-alpha (panel e) levels in the medium were determined by ELISA. The results were compared by the Kruskal-Wallis test with the Holm-Sidak posttest or Student’s t test

HDL was utilized as an acceptor of ^14^C-cholesterol from BMDMs as a tool to determine the intrinsic capacity of HDL to mediate cholesterol efflux. As shown in Fig. 4 (panel a), ^14^C-cholesterol efflux mediated by HDL isolated from the eGFR < 60 + A3 group was lower than that mediated by HDL from the control group. The antioxidant activity of HDL that minimizes LDL oxidation was determined by measuring LDL oxidation with CuSO_4_ over time. The lag phase for LDL oxidation determined by the presence of HDL was similar among all groups (Fig. 4 panel b), as was the maximum ratio of conjugated diene formation in LDL (Fig. 4 panel c). Additionally, the ability of HDL from subjects with DKD and controls to inhibit the secretion of inflammatory cytokines by LPS-challenged cells was measured. Figure 4 (panel d and e) shows the secretion of the inflammatory cytokines IL-6 **(panel d)** and TNF-alpha **(panel e)** in macrophages treated with HDL from the controls and patients with DKD with eGFR < 60 + A3 group and after challenge with LPS. For both cytokines, a very lower secretion was observed when macrophages were exposed to DKD HDL in comparison to C-HDL.

**Fig. 5:**
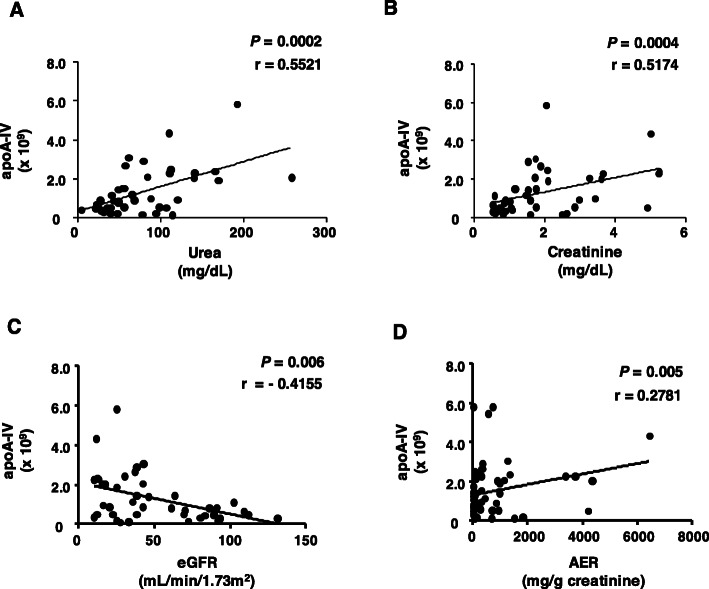
Correlations between apoA-IV and CKD parameters. Correlations were performed using Spearman correlation

**Fig. 6:**
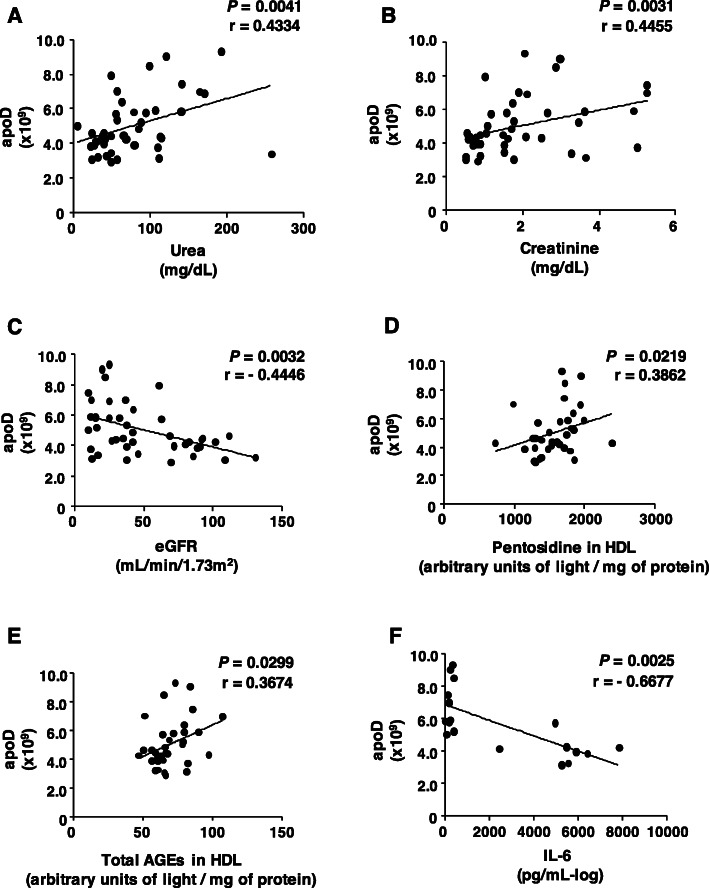
Correlations between apoD and parameters of CKD, HDL chemical modification and functionality. Correlations were performed using Spearman correlation

ApoA-IV in the HDL proteome was positively correlated with urea **(**Fig. 5 panel a), creatinine (Fig. 5 panel b), and AER (Fig. 5 panel d) and inversely correlated with eGFR **(**Fig. 5 panel c). No correlation was observed between apoA-IV and any measured parameters of HDL functionality or HDL chemical modification (data not shown). ApoD was also positively correlated with urea **(**Fig. 6 panel a) and creatinine urea **(**Fig. 6 panel b**)** and inversely correlated with eGFR **(**Fig. 6 panel c**)**, although no correlation was observed with AER (data not shown). A positive correlation was observed between apoD and pentosidine and total AGEs in HDL **(**Fig. 6 panels d and e**)**. In addition, apoD was negatively correlated with the secretion of IL-6 **(**Fig. 6 panel f**)** but not associated with TNF-alpha (data not shown).

## Discussion

The prevalence of kidney complications in DM is high, and the AER together with the reduced eGFR independently predicts CVD morbidity and mortality [19]. Changes in the HDL proteome and functionality may modulate the antiatherogenic actions of this lipoprotein and consequently the development of atherosclerosis [20]. In this study, the composition, chemical modification and proteomics of HDL particles and their ability to remove cholesterol from macrophages and inhibit LDL oxidation and macrophage inflammation were evaluated in patients with DM2 with DKD.

Targeted proteomics quantified 28 proteins in HDL of all experimental groups, although only 2 – apoD and apoA-IV – were differentially expressed in DKD with eGFR < 60 plus A3. ApoD is an atypical apolipoprotein mainly expressed in the central nervous system, testes, adrenal glands and kidneys [21]. Its expression is elevated by aging [22] and is implicated in neurological [23] and psychiatric [24] disorders. In mice, apoD protects against oxidative stress, which is related to its ability to scavenge free radicals [25]. In HDL, this apolipoprotein contributes to HDL hydrophobic nucleus remodeling by facilitating lecithin-cholesterol acyltransferase (LCAT) anchoring to the lipoprotein structure and carrying lysophosphatidylcholine, although it is not clear whether it has the potential to activate or inhibit the enzyme [26]. Some studies have shown an increase in apoD in the HDL proteome of individuals with coronary artery disease and in areas of human atherosclerotic lesions as well as in apoE knockout mouse plasma [26–28]. However, it is not yet clear whether this increase in apoD in these conditions refers to its role in inducing atherosclerosis or whether it represents a compensatory adaptive mechanism to changes observed in cardiovascular disease [26]. In the present investigation, apoD was related for the first time to markers of kidney failure (including urea, creatinine, and eGFR). In addition, apoD was positively correlated with HDL advanced glycation and negatively related to the secretion of IL-6 by LPS-challenged macrophages that were treated with HDL.

Several studies have demonstrated that apoA-IV can be used as an early marker of kidney failure in individuals with CKD and in the general population, although further studies are needed to understand the pathophysiological basis of this association [29]. In addition, in individuals on hemodialysis, the increase in apoA-IV was associated with an increased risk of all-cause mortality [30]. The results from the present investigation agree with these findings, and increased apoA-IV expression was observed in the HDL proteome of individuals with higher AER and reduced eGFR compared to controls. Additionally, apoA-IV was positively correlated with urea, creatinine, and AER and inversely correlated with eGFR. This apolipoprotein is synthesized in the intestine and secreted in the mesenteric lymph, being transported by chylomicrons but mainly by HDL, and is the third most abundant apolipoprotein in this lipoprotein [29, 30]. ApoA-IV presents many antiatherogenic functions, acting in the removal of cellular cholesterol [31] and promoting the activation of lipoprotein lipase [32], LCAT and cholesteryl ester transfer protein (CETP) [33]. In addition, its antiatherogenic activity is complemented by its anti-inflammatory and antioxidant properties [34, 35]. Nonetheless, in the present investigation, apoA-IV was not correlated with cytokine secretion by macrophages treated with HDL or with other HDL functions (cholesterol efflux and inhibition of LDL oxidation). Future experiments with apoA-IV enrichment or deletion in HDL particles would help to clarify its involvement in CVD protection in DKD.

Similar to other HDL apolipoproteins, apoA-IV is modified by advanced glycation in DM and CKD. Recently, it was demonstrated that *E. coli* recombinant apoA-IV submitted to advanced glycation in vitro maintains its ability to remove excess macrophage cholesterol, despite its large amounts of pyrraline, CML and argypyrimidine [36]. This may explain why no major reductions in cholesterol efflux were observed in the present study, even with a significant increase in total AGEs and pentosidine in the HDL of the eGFR < 60 + A3 group compared to controls. In addition, the in vitro glycation of apoA-IV only partially impaired its ability to inhibit the inflammatory response promoted by LPS in macrophages [37]. In contrast, apoA-I has the ability to remove cell cholesterol, and antioxidant and anti-inflammatory properties are severely impaired by advanced glycation [29].

The HDL proteome has been analyzed in CKD, but the vast majority of studies were performed in individuals undergoing hemodialysis, which can interfere with the observed results. Additionally, many studies included CKD together with DKD indistinctively. From 120 proteins detected, Mangé et al. [38] found 40 differentially expressed proteins in HDL comparing healthy controls and nondiabetic subjects with CKD on dialysis, including apoA-IV and apoD, which were upregulated in individuals undergoing hemodialysis. Nonetheless, the authors only discussed the meaning of apoC-II and apoC-III, which were higher while transtirretin was lower in HDL from subjects with CKD on hemodialysis compared to healthy subjects in an independent population that was used to validate proteomic analyses. In another study, 35 proteins were detected in HDL from individuals on hemodialysis, with or without DM, and healthy controls. ApoA-IV was only detected in uremic HDL and was positively correlated with HDL-associated albumin, while apoD was found in all samples from both groups. Only SAA1, albumin, phospholipase A2 and apoC-III were differentially expressed between HDL from controls and those with uremia. Along with the reduction in the content of phospholipids and an increase in TG and lysophospholipids, these modifications were linked to the reduction in HDL-mediated cholesterol efflux [39]. In agreement, HDL dysfunction was also related to changes in the HDL proteome by Florens et al. [40], although apoA-IV and apoD were not included among the 19 proteins that were up- or downregulated in nondiabetic patients on hemodialysis in comparison to controls. Shao et al. [41], by utilizing shotgun proteomics followed by the quantification of HDL proteins by isotope dilution and selected reaction monitoring, found 22 proteins upregulated in stage 5 CKD, including apoA-IV, and 6 downregulated proteins. Nonetheless, there was no mention of the intrinsic HDL functionality. Many of the proteins that increased in HDL of individuals with CKD were related to renal injury (beta 2 microglobulin, complement factor D, cystatin C, prostaglandin-H2 D-isomerase, retinol-binding protein 4 and AMBP). Others were more present in the control group, among them apoA-I, apoA-II, apoL-I, apoM, and paraoxonase 1 (PON-1), probably conferring greater damage to the antiatherogenic activities of HDL in CKD.

Recently, the HDL proteome was analyzed in 191 individuals with DM1 in the Diabetes Control and Complications Trial (DCCT) study. Eight proteins were associated with proteinuria, although only PON-1 was simultaneously associated with AER and coronary calcium content [42]. Wang et al. [43] demonstrated enrichment of 8 proteins related to inflammation and lipid metabolism (serum amyloid A1, A2, and A4; hemoglobin beta; HPTR; CETP; PLTP; and apoE) in HDL isolated from individuals in the short term compared to long-term dialysis therapy. It is not clear how dialysis may change the HDL proteome and composition in DKD. One aspect is the competition between carbamoylation and glycation reactions; dialysis removes urea, limiting carbamoylation, but does not remove AGEs or AGE precursors that react with free lysine residues in proteins [44]. In addition, according to the dialytic procedure, small AGE precursors can be removed or added to lipoproteins, namely, in the case of peritoneal dialysis. Then, dialysis may differentially affect carbamoylation and glycation and even inflammation. Regarding proteomics, it is not known how dialytic procedures affect the HDL proteome, especially the HDL cargo in small peptides that can easily dissociate from HDL, affecting its function and structure.

The proteomic analysis utilized in the present investigation offered a relative quantification of apoA-I since no internal standard for this apolipoprotein was used in this investigation. Although not statistically different among groups, apoA-I in the HDL proteome was inversely correlated with the AER (r = − 0.5549; *P* = 0.0005). In the efflux assay performed in this study, the HDL concentration was matched by the protein component that is mainly represented by apoA-I, although in vivo apoA-I can be dissociated from the HDL particle and this may affect cholesterol efflux. A decrease in HDL phospholipids was observed in the eGFR < 60 + A3 group, with a positive correlation with eGFR. Notably, phospholipids are positive modulators of cell cholesterol removal, as they facilitate the interaction of HDL with plasma membrane domains that guarantee cholesterol exportation by diffusion and/or mediated by specific receptors.

A reduced ability to remove cell cholesterol was previously reported in individuals with stage 3 and 4 CKD compared to healthy controls, although this was not independent of age [45]. In this sense, there are studies showing the role of aging on the ability of HDL to mediate cholesterol efflux, which may be a bias for many studies dealing with controls and subjects with CKD at different ages [46, 47]. Yamamoto et al. [48] reported a reduced cholesterol efflux mediated by HDL from individuals on dialysis with DM with or without DKD in comparison to controls.

Differently from the present work, there are many studies that measured cholesterol efflux mediated by apoB-depleted serum. Although HDL is the only lipoprotein in that serum, there are many other components, including albumin, cytokines, haptoglobin and insulin, that could affect cholesterol removal. In addition, measurement of cholesterol removal mediated by apoB-depleted serum hides the variations in HDL cholesterol levels that are frequently altered in DM and DKD [49, 50].

Ganda et al. [51] did not find any alteration in cholesterol efflux mediated by apoB-depleted serum isolated from subjects with CKD (stages 4 and 5), although the expression of *ABCA1 *in monocytes isolated from those subjects was reduced, compromising cholesterol efflux to apoA-I. Chemical modification of HDL by advanced glycation and carbamoylation that was observed in the present study was related to the stage of AER and eGFR. In individuals undergoing hemodialysis or peritoneal dialysis, a greater amount of pentosidine was found in plasma that correlated with the progression of kidney disease. In addition, pentosidine levels were higher in CKD stage 5 subjects than in stage 1 subjects [52]. Pentosidine and other AGEs found to be elevated in HDL from subjects included in the present investigation are consequences of hyperglycemia that is inherent to DM as well as failure in glycation intermediate detoxification due to CKD. These conditions simultaneously contribute to AGE generation, which can be further aggravated by inflammation and exogenous sources. In subjects with CKD undergoing hemodialysis, an elevated concentration of carbamoylated HDL was demonstrated [53, 54]. Both glycation and carbamoylation have a negative impact on atherogenesis by favoring the uptake of LDL to the detriment of the HDL-mediated cholesterol efflux that is impaired by these modifications [55, 56].

The antioxidant capacity of HDL assessed by the lag time for LDL oxidation was similar among groups, and as for the other HDL functions, the impact of apoD and apoA-IV in the HDL proteome should be investigated by using appropriate experimental protocols. For instance, the antioxidant role of HDL can be assessed by using cell-based models that sometimes show divergent results compared to the measurement of the lag phase utilized in the present investigation.

Interestingly, the anti-inflammatory ability of HDL was greatly increased in DKD-HDL compared to control HDL. This was demonstrated by the reduced secretion of the inflammatory cytokines IL-6 and TNF-alpha by macrophages challenged by LPS.

## Study strengths and limitations

This is the first study dealing specifically with subjects with DKD without dialysis and categorized by eGFR and AER to show a difference in the HDL proteome that relates to its function. The limitations relate to the small number of individuals included due to the exclusion criteria. In addition, although there is no direct evidence of a cause-effect relationship, the findings point to apoD and apoA-IV as markers of DKD that may contribute to HDL function, despite lipoprotein chemical modification by carbamoylation and advanced glycation, which deserves future investigation.

## Conclusion

Enhanced apoD and apoA-IV contents in the HDL proteome in subjects with DKD without dialysis was demonstrated, which may modulate the antiatherogenic functions of this lipoprotein. The increment in apoD and apoA-IV could contribute to counteracting the HDL chemical modification by AGEs and carbamoylation that contributes to HDL loss of function in well-established DKD and should be further investigated.

## Data Availability

The dataset used and/or analyzed during the current study is available from the corresponding author upon reasonable request. The authors declare that all data generated or analyzed during this study are included in this published article [and its supplementary information files].

## Abbreviations

apoA-I: Apolipoprotein A-I
apoA-IV: Apolipoprotein A-IV
apoC-IV: Apolipoprotein C-IV
apoD: Apolipoprotein D
AER: Albumin excretion rate
AGEs: Advanced glycated end products
BMDM: Bone marrow-derived macrophages
CKD: Chronic kidney disease
CVD: Cardiovascular disease
DKD: Diabetic kidney disease
eGFR: Estimated glomerular filtration rate
HDL: High-density lipoprotein
LDL: Low-density lipoprotein
RCT: Reverse cholesterol transport
SAA4: Serum amyloid protein A-4
